# Maternal metabolic syndrome in pregnancy and child development at age 5: exploring mediating mechanisms using cord blood markers

**DOI:** 10.1186/s12916-023-02835-5

**Published:** 2023-04-03

**Authors:** Janell Kwok, Lydia Gabriela Speyer, Georgia Soursou, Aja Louise Murray, Kostas A. Fanti, Bonnie Auyeung

**Affiliations:** 1 School of Philosophy, Psychology and Language Sciences, University of Edinburgh, 7 George Square, Edinburgh, EH8 9JZ UK; 2 Department of Psychology, University of Cambridge, Cambridge, UK; 3 Center for Applied Neuroscience, University of Cyprus, Nicosia, Cyprus; 4 Department of Psychology, University of Cyprus, Nicosia, Cyprus; 5 Autism Research Centre, Department of Psychiatry, University of Cambridge, Cambridge, UK

**Keywords:** Metabolic, Prenatal, Pregnancy, Biomarkers, Child development, Born in Bradford, Cord blood, Mediation analysis

## Abstract

**Background:**

There is limited evidence on how the classification of maternal metabolic syndrome during pregnancy affects children’s developmental outcomes and the possible mediators of this association. This study uses a cohort sample of 12,644 to 13,832 mother–child pairs from the UK Born in Bradford Study to examine the associations between maternal metabolic syndrome classification (MetS) and child development outcomes at age 5, using cord blood markers as candidate mediators.

**Methods:**

Maternal cardiometabolic markers included diabetes, obesity, triglycerides, high-density lipoprotein cholesterol, blood pressure, hypertension, and fasting glucose during pregnancy. Cord blood markers of high-density lipoprotein cholesterol, low-density lipoprotein cholesterol, triglycerides, leptin, and adiponectin were used as child mediators. Child outcomes included two starting school variables: British Picture Vocabulary Scale (BPVS) and the Letter Identification Assessment (LID), and five developmental milestone domains from a national UK framework: (1) communication and language (COM); (2) personal, social, and emotional (PSE); (3) physical development (PHY); (4) literacy (LIT); and (5) mathematics (MAT). Mediation models were used to examine the associations between the classification of maternal metabolic syndrome and child developmental milestones. Models were adjusted for potential maternal, socioeconomic, and child confounders such as maternal education, deprivation, and gestational age.

**Results:**

In mediation models, significant total effects were found for MetS associations with children’s development in the LIT domain at age 5. MetS predicted individual cord blood mediators of lower HDL and increased leptin levels in both adjusted and unadjusted models. Total indirect effects (effects of all mediators combined) for MetS on a child’s COM and PSE domain were significant, through all child cord blood mediators of LDL, HDL, triglycerides, adiponectin, and leptin for adjusted models.

**Conclusions:**

The results support the hypothesis that maternal metabolic syndrome classification during pregnancy is associated with some child developmental outcomes at age 5. After adjusting for maternal, child, and environmental covariates, maternal metabolic syndrome classification during pregnancy was associated with children’s LIT domain through direct effects of maternal metabolic health and indirect effects of cord blood markers (total effects), and COM and PSE domains via changes only in a child’s cord blood markers (total indirect effects).

**Supplementary Information:**

The online version contains supplementary material available at 10.1186/s12916-023-02835-5.

## Background

There is limited evidence from human studies on how maternal cardiometabolic health during pregnancy affects a child’s cognitive and behavioural development. While it has been established that a mother’s metabolic state during gestation plays an essential role in foetal early-life programming, less is known about the subsequent effects of this programming on a child’s development beyond immediate birth outcomes [1]. Emerging evidence on individual maternal cardiometabolic factors such as BMI, glucose, cholesterol levels [2], and blood pressure [3] have been shown to possibly alter the key signalling pathways for brain development through energy metabolisation and foetal cell growth modulation. Furthermore, dysfunction in foetal programming is linked with higher susceptibility for immune function and cardiovascular disease from childhood and even into adolescence [4]. This dysfunction in turn is suspected to affect not only birth and health outcomes [5, 6], but also a child’s neurodevelopment [7]. The presence of maternal cardiometabolic risk in pregnancy has been found to be associated with executive functioning, impulse control behaviour [8], language skills [9], psychomotor development [10], communication [11], and mental health [12] in their children.

Cardiometabolic risk factors often occur in a cluster rather than independently [13] due to multifactorial interactions [13]. Biologically, the co-occurrence of these factors presents as more intrauterine stress and subsequent inflammatory downstream responses [14]. Maternal metabolic syndrome in pregnancy, also classified as cardiometabolic risk, is associated with more adverse pregnancy and birth complications [15]. Metabolic syndrome is a constellation of risk factors for developing cardiovascular disease, diabetes, and obesity. Raised glucose levels, high cholesterol, high blood pressure, and increased body mass index (BMI) are classified as measures of metabolic syndrome. Components such as hyperglycaemia and related changes in blood lipids (increase in triglycerides and decrease in HDL) further increase a person’s risk of having metabolic syndrome. The World Health Organization (World Health Organization, 2016), the European Group for the Study of Insulin Resistance (EGIR), and the National Cholesterol Education Program – Third Adult Treatment Panel presented several core components of the metabolic syndrome: obesity, insulin resistance, dyslipidaemia, and hypertension [16, 17].

Recent evidence has also found maternal metabolic syndrome during pregnancy to be associated with shortened telomere length in offspring, showing cell tissue damage which increases future disease risk [18]. While shortened telomere length as a result of oxidative stress has also been associated with a predisposition to future mood disorders [19], there is still much unknown on the mechanistic effects of metabolic syndrome on offspring neurodevelopment [20], showing the need to understand these associations as early on as possible.

Despite evidence for links between maternal metabolic health profiles and child outcomes, there is a gap in understanding the biological pathways underlying the association. An under-explored area in human studies is the maternal-newborn biological interface. Using cord blood markers as proxies of a child’s cardiometabolic health status, temporal associations can then potentially be drawn where the newborn has not yet been directly exposed to other environmental influences, such as education or peer relationships. This is crucial for separating gestational biology and environmental influences and can potentially be a biomarker for a child’s eventual neurodevelopmental trajectory [21], leading to important clinical implications such as medical interventions during pregnancy or early monitoring at the start of formal education for the child.

Standardised developmental assessments are important for closer examination of a child’s development when examining practical outcomes. The British Picture Vocabulary Scale (BPVS), Letter Identification Assessment (LID), and domains within Early Years Foundation Stage (EYFS) statutory framework are examples of such developmental milestones expected of a child. They are assessed by early year school providers in England through classroom observations at the end of the school year by the time a child turns 5 years old [22]. Fulfilling the EYFS framework is a standard set by the government, for early year providers to assess how children learn and develop well, and possess the skills required to start school, which can be a precursor for future cognitive development and educational attainment [22], and therefore relevant to this study.

This study aims to examine the associations between maternal metabolic syndrome and child development outcomes at 5 years old and whether child metabolic health at birth using cord blood markers mediates the association between maternal metabolic health and child development outcomes, while also accounting for potential confounders such as maternal smoking and alcohol intake during pregnancy or child gestational age. This study’s hypotheses are (a) maternal metabolic syndrome classification during pregnancy is associated with lowered scores in children’s developmental domains at 5 years old and that (b) child cord blood markers mediate the association between maternal metabolic syndrome and a children’s developmental scores at 5 years old.

## Methods

### Study participants

The Born in Bradford (BiB) Study is a longitudinal multi-ethnic birth cohort study aiming to examine the impact of environmental, psychological, and genetic factors on maternal and child health and well-being [23]. Bradford is a city in the North of England with high levels of socioeconomic deprivation and ethnic diversity. Women were recruited at the Bradford Royal Infirmary at 26–28 weeks of gestation. For those consenting, a baseline questionnaire was completed. The full BiB cohort recruited 12,453 women and 3353 of their partners across 13,776 pregnancies and 13,858 children between 2007 and 2010. The cohort is broadly characteristic of the city’s maternal population. Ethical approval for the data collection was granted by Bradford Research Ethics Committee (Ref 07/H1302/112). Full details on recruitment, attrition, and assessment procedures in BiB can be found on the study website (https://borninbradford.nhs.uk/). Of the total sample, singleton births made up *n* = 13,455, while twins made up *n* = 354, triplets made up *n* = 9, and unknown for *n* = 40. As the proportion of multiple births was not substantial, this study’s mediation models did not consider these effects.

### Measures

#### Cardiometabolic measures

Data on cardiometabolic measures were taken from the mother’s baseline questionnaire, pregnancy blood biomarkers, and pregnancy data from electronic records. Continuous variables taken from the mother’s baseline questionnaire dataset included the mother’s body mass index (BMI) (12.9–57.0) and triglycerides (mmol/L; 0.6–17.8). Continuous variables taken from the pregnancy data from electronic records, backfilled notes, or pregnancy blood biomarkers included systolic blood pressure at 28 weeks (mmHg; 52–188), diastolic blood pressure at 28 weeks (mmHg; 35–114), fasting glucose (mmol/L; 3.0–13.3), and HDL (mmol/L; 0.6–4.1). Categorical variables included existing hypertension (yes or no) and diabetes prior to pregnancy (yes or no). Descriptive information for all maternal cardiometabolic markers is available in Table 1. More information can be found in the BIB cohort profile paper [23].

**Table 1:** Descriptive statistics for predictors

**Categorical variables**	**Number**	**Per cent**	
**Metabolic syndrome classification**			
**Yes**	537	4.9	
No	10,495	95.1	
**BMI**			
≥ **30**	2248	21.2	
< 30	8379	78.8	
**Triglycerides (mmol/L)**			
** > 1.7**	6775	57.9	
≤ 1.7	4920	42.1	
**HDL (mmol/L)**			
≤ **1.6**	1864	15.9	
> 1.6	9831	84.1	
**Systolic BP (mmHg)**			
≥ **140**	180	1.5	
< 140	12,162	98.5	
**Diastolic BP (mmHg)**			
≥ **90**	62	0.5	
< 90	12,281	99.5	
**Fasting glucose (mmol/L)**			
> **5.6**	387	3.0	
≤ 5.6	12,016	96.9	
**Hypertension (previously diagnosed)**			
**Yes**	117	0.9	
No	12,808	99.1	
**Previously diagnosed diabetes**			
**Yes**	350	3.0	
No	11,235	97.0	
**Continuous variables**	**Mean (SD)**	**Median (IQR)**	**Range**
**BMI (*n* = 10,627)**	26.04 (5.7)	25.04 (21.93–29.10)	12.9–57
**Triglycerides (mmol/L) (*n* = 11,695)**	1.98 (0.74)	1.90 (1.50–2.30)	0.6–17.8
**HDL (mmol/L) (*n* = 11,695)**	1.97 (0.43)	1.90 (1.70–2.20)	0.6–4.1
**Systolic BP (mmHg) (*n* = 12,342)**	109.53 (11.51)	110.0 (100.0–120.0)	52–188
**Diastolic BP (mmHg) (*n* = 12,343)**	64.87 (8.34)	64.0 (60.0–70.0)	35–114
**Fasting glucose (mmol/L) (*n* = 12,403)**	45.3 (0.55)	4.40 (4.20–4.70)	3–13.3

*SD* standard deviation, *IQR* interquartile range

#### Maternal metabolic syndrome classification

In this study, maternal metabolic syndrome is classified according to the International Diabetes Federation definition: maternal BMI of > 30 kg/m^2^, together with two of the following factors: raised triglycerides (≥ 1.7 mmol/L), reduced HDL cholesterol (< 1.29 mmol/L), raised blood pressure (systolic BP ≥ 130 or diastolic BP ≥ 85 mmHg or treatment of previously diagnosed hypertension), raised fasting plasma glucose (≥ 5.6 mmol/L), or previously diagnosed type 2 diabetes [24].

#### Child cardiometabolic mediators

Cord blood markers are commonly used as a clinical evaluation of newborn health and were selected to be candidate child mediators for this study. In addition to previously selected lipid markers to match maternal metabolic markers, hormones of adiponectin and leptin were selected due to emerging literature on the effects on infants’ cognition between 6 and 24 months and IQ levels and working memory between ages 3 and 8 years old [25]. All child cardiometabolic markers were continuous variables. Child markers in this study included low-density lipoprotein (LDL), high-density lipoprotein (HDL), triglycerides, adiponectin, and leptin levels as taken from blood assays at birth. Descriptive information for all child cardiometabolic markers is available in Table 2.

**Table 2:** Descriptive statistics for child mediators and developmental outcomes

**Child mediators**	**Mean (SD)**	**Median (IQR)**	**Range**
**HDL (mmol/L)**	0.66 (0.22)	0.62 (0.50–0.78)	0.09–1.66
**LDL (mmol/L)**	0.80 (0.28)	076 (0.613–0.940)	0.02–3.21
**Triglycerides (mmol/L)**	0.53 (0.26)	0.46 (0.36–0.62)	0.18–2.64
**Adiponectin (ng/ml)**	31.68 (13.32)	29.8 (22.5–38.5)	2.6–98.1
**Leptin (ng/ml)**	10.53 (12.18)	7.11 (3.79–13.21)	0.15–430.87
**Child outcomes**	**Mean (SD)**		**Range**
**Age at assessment (months)**	59.6 (1.00)	60.0 (56.0–63.0)	56–61
** Starting school variables**			
British Picture Vocabulary Scale (*n* = 3293)	100.75 (15.66)	100.0 (92.0–110.0)	39–161
Letter identification (*n* = 3259)	106.45 (12.60)	106.0 (97.0–117.0)	68–143
** EYFS childhood outcomes (*n* = 10,600)**			
COM: communication and language	5.93 (1.77)	6.0 (5.5–6.0)	3–9
PSE: personal, social and emotional	5.91 (1.54)	6.0 (6.0–6.0)	3–9
PHY: physical development	4.02 (1.00)	4.0 (4.0–4.0)	2–6
LIT: literacy	3.57 (1.24)	4.0 (2.0–4.0)	2–6
MAT: mathematics	3.61 (1.16)	4.0 (2.0–4.0)	2–6

Sample sizes are dependent on the availability of predictors, outcomes, mediators, covariates and confounders

*SD* standard deviation, *IQR* interquartile range

### Development outcomes

#### Starting school variables

Starting school measures included the British Picture Vocabulary Scale (BPVS) [26] and the Letter Identification assessment (LID) by the Born in Bradford team. The BPVS is a one-to-one test assessment of a child’s receptive vocabulary. It is used to assess language development in non-readers or students with communication difficulties or expressive language impairments. The LID is a school-based assessment used by teachers to test for the core skill of identifying letters and sounds at school age.

#### Early years foundation stage framework

The Early Years Foundation Stage (EYFS) statutory framework was included to assess the developmental milestone outcomes. Each EYFS domain was categorised as ‘1 = emerging’, ‘2 = expected’, ‘3 = exceeding’, and ‘4 = absent for long periods or recently arrived’ per measure. In this study, ‘4 = absent for long periods or recently arrived’ was coded as missing. The number of children that fell in this category was *n* = 9, and only 1–3 were analysed for the scale to show developmental ability. Providers are to indicate on the EYFS profile whether children are meeting the expected levels of development, are exceeding expected levels, or have not yet reached expected levels (‘emerging’). This study included five domains from this developmental framework to cover a range of essential developmental skills for a child at 5 years of age.

- COM: communication and language development (listening and attention, understanding, and speaking)
- PSE: personal, social, and emotional development (self-confidence and self-awareness, management of feelings and behaviour, making relationships)
- PHY: physical development (moving and handling, and health and self-care)
- LIT: literacy (reading and writing)
- MAT: mathematics (numbers, shapes, space, and measures)

For all child outcomes, mother–child pair participant samples were *n* = 12,644 to *n* = 13,364 for the unadjusted models and *n* = 13,812 to 13,832 for the adjusted models based on the availability of data, with no children excluded from the analysis. Descriptive information for the starting school variables and developmental domains on childhood outcomes is available in Table 2.

#### Confounders and covariates

Possible confounders included maternal and socioeconomic factors as obtained by the research team. Previous studies have acknowledged important potential confounders such as parental education, maternal smoking during pregnancy, socioeconomic status, and gestational age when examining maternal cardiometabolic markers and childhood outcomes [17, 27, 28]. Maternal age was coded as a continuous variable. Dichotomous variables included maternal education being coded into A’ levels and higher or lower than A’ levels, previous alcohol intake as yes or no, and previous smoking as yes or no. The deprivation index in this study was taken from the official United Kingdom Index of Multiple Deprivation, where area-level deprivation was measured which accounts for domains of deprivation (employment, income, health, education, housing, and living environment), and was already sorted into quintile ranks of 1–5, with 1 being the lowest deprivation and 5 being the highest deprivation exposure. Additional covariates included maternal ethnicity, child sex, gestational age, and birth weight. Maternal ethnicity was coded as ‘White British’, ‘Pakistani’, and ‘Others’. Child sex was coded as male or female. Gestational age was used as a continuous variable in the measurement of weeks, and birth weight was coded as a continuous variable in the measurement of kilogrammes. The fully adjusted model included possible confounders of maternal education, maternal age, maternal alcohol intake, maternal smoking, deprivation indices, and the additional covariates of maternal ethnicity, child sex, gestational age, and birth weight. The descriptive information for all study confounders and covariates is available in Table 3.

**Table 3:** Descriptive statistics for confounders and covariates

	**MetS classification (*n* = 537)**		**No MetS classification (*n* = 10,495)**	
	**Mean (SD)/*N* (%)**	**Range**	**Mean (SD)/*N* (%)**	**Range**
**Confounders**				
**Maternal age**	29.33 (5.86)	16–45	27.14 (5.55)	15–49
**Maternal education**				
≥ A’ levels	146 (29.7%)		3887 (44.2%)	
< A’ levels	345 (70.3%)		4898 (55.8%)	
**Maternal alcohol history**				
Yes	180 (33.5%)		2915 (30.6%)	
No	357 (66.5%)		6615 (69.4%)	
**Maternal smoking history**				
Yes	114 (21.2%)		1546 (16.2%)	
No	423 (78.8%)		7992 (83.3%)	
**Index of multiple deprivation**				
80–100%	384 (71.5%)		6276 (65.7%)	
60–80%	85 (15.8%)		1739 (18.2%)	
40–60%	46 (8.6%)		1083 (11.3%)	
20–40%	16 (2.9%)		280 (2.9%)	
0–20%	6 (1.2%)		172 (1.9%)	
**Covariates**				
**Maternal ethnicity**				
White British	310 (52.1%)		5229 (47.6%)	
Pakistani	225 (37.8%)		4309 (39.2%)	
Others	60 (10.1%)		1455 (13.2%)	
**Child sex**				
Male	274 (51.0%)		5087 (48.5%)	
Female	263 (48.9%)		5407 (51.5%)	
**Child gestational age (weeks)**	38.7 (2.02)	27–42	39.1 (1.82)	25–44
**Child birthweight (kg)**	3.31 (0.67)	0.67–5.7	3.21 (0.56)	0.48–5.32

*SD* standard deviation, *IQR* interquartile range

### Statistical analysis

Age and child sex were raw standard scores. The other variables were standardised for analysis to facilitate estimation by putting variables on similar scales. A structural equation model was used to test the mediating role of child cardiometabolic markers of LDL, (HDL), triglycerides, adiponectin, and leptin in relationships between the maternal metabolic syndrome construct and child developmental outcomes (BPVS, LID, COM, PSE, PHY, LIT, MAT). Correlations were run for child cardiometabolic variables (HDL, LDL, triglycerides, adiponectin, leptin) and study covariates. A mediation model was specified using the maternal metabolic syndrome construct as the predictor (*X*), with child cardiometabolic markers as the mediators (*M*) and developmental assessments as the outcome (*Y*). This study defined direct effects as effects of *X* on *Y* after adjustment for indirect effects, indirect effects as effects of *X* on *Y* transmitted through *M*, and total effects of as the sum of all effects of *X* on *Y* (both indirect and direct), and total indirect effects as the sum of all indirect effects [29]. Direct, indirect, total indirect, and total (direct + indirect) effects on child development outcomes were modelled, accounting for multiple mediators [30]. Mediation proportions were derived using the ratio of the mediation effect estimate and the total effect estimate.

Models were estimated using a robust estimator to account for the non-normal distribution of the outcome. Missing data was handled using full information maximum likelihood estimation. To evaluate the statistical significance of mediation effects using bootstrapped confidence intervals, we also ran bootstrapped models with standard maximum likelihood estimation. Models were fit using the MPlus statistical software (version 8.7) [31].

**Fig. 1:**
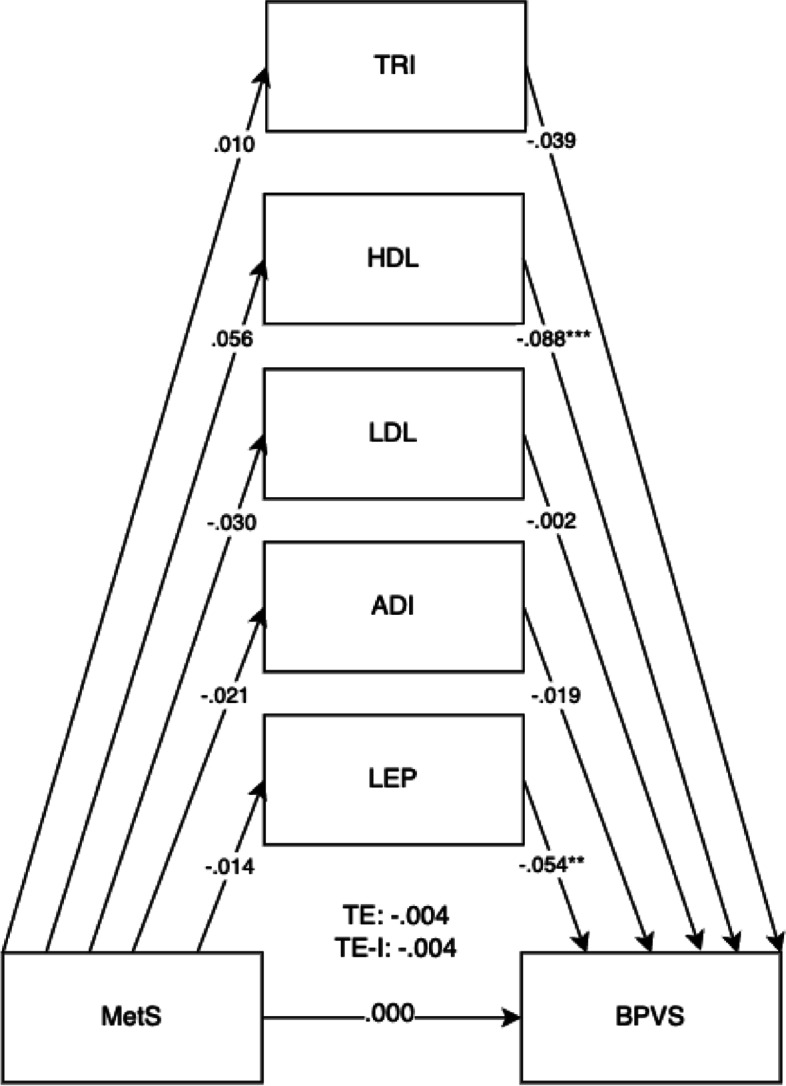
Mediation model for BPVS outcomes at 5 years old with cord blood mediators of low-density lipoprotein (LDL), adiponectin (ADI), leptin (LEP), triglycerides (TRI), and high-density lipoprotein levels (HDL). Adjusted for potential covariates of maternal education, maternal age, maternal alcohol intake, maternal smoking, deprivation indices, child sex, ethnicity, gestational age, and birth weight. TE, total effect (direct effect + indirect effect); T-IE, total indirect effect. *p < .05, **p < .01, ***p < .001

**Fig. 2:**
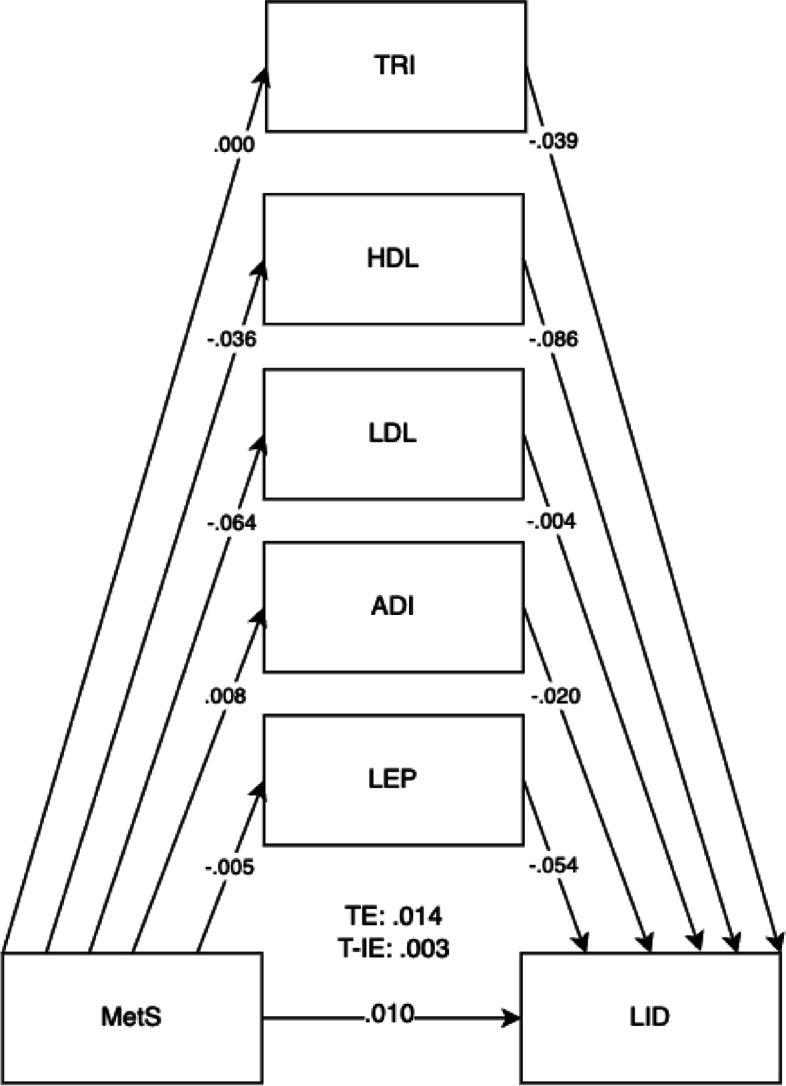
Mediation model for LID outcomes at 5 years old with cord blood mediators of low-density lipoprotein (LDL), adiponectin (ADI), leptin (LEP), triglycerides (TRI), and high-density lipoprotein levels (HDL). Adjusted for potential covariates of maternal education, maternal age, maternal alcohol intake, maternal smoking, deprivation indices, child sex, ethnicity, gestational age, and birth weight. TE, total effect (direct effect + indirect effect); T-IE, total indirect effect. *p < .05, **p < .01, ***p < .001

**Fig. 3:**
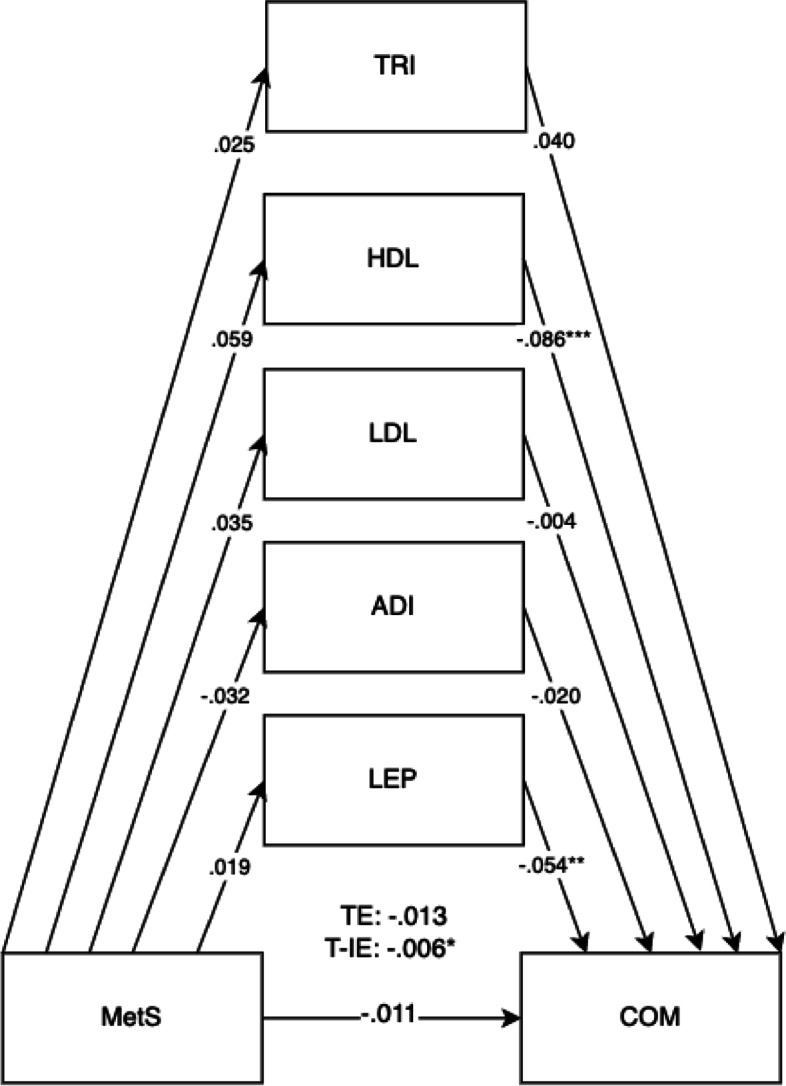
Mediation model for MetS on child communication and language outcomes (COM) at 5 years old with cord blood mediators of low-density lipoprotein (LDL), adiponectin (ADI), leptin (LEP), triglyceride (TRI)s, and high-density lipoprotein levels (HDL) levels. Adjusted for covariates of maternal education, maternal age, maternal alcohol intake, maternal smoking, deprivation indices, child sex, ethnicity, gestational age, and birth weight. TE, total effect (direct effect + indirect effect); T-IE, total indirect effect. *p < .05, **p < .01, ***p < .001

**Fig. 4:**
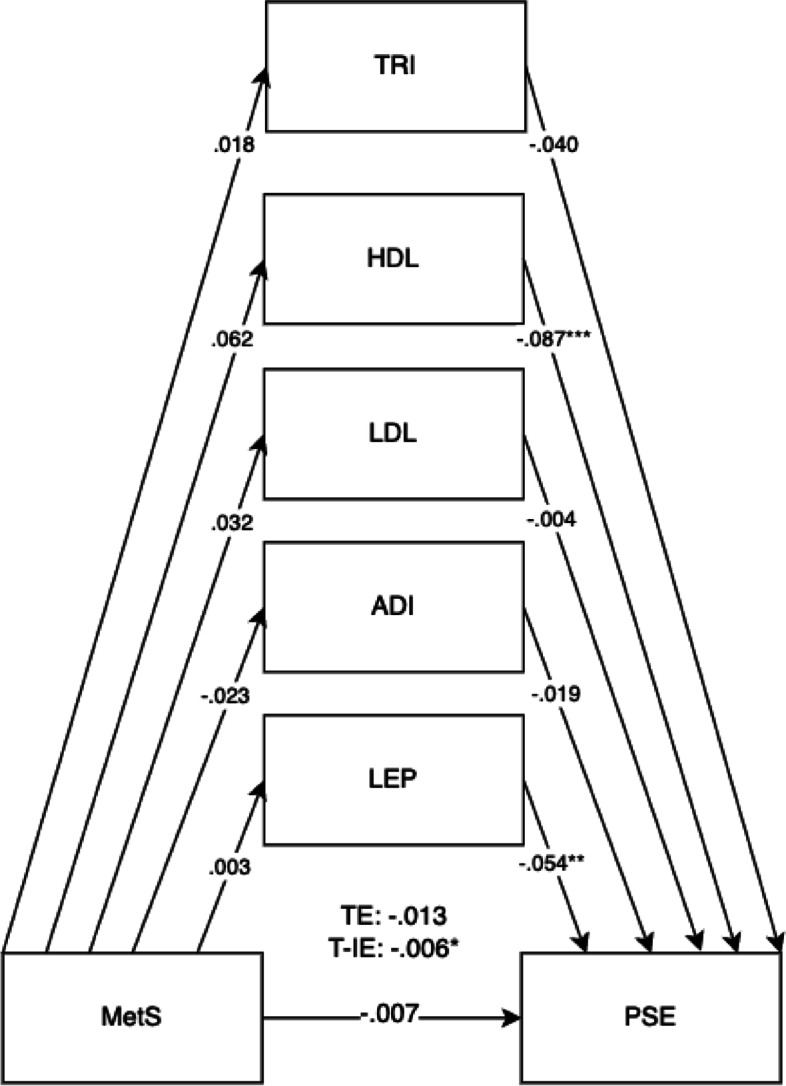
Mediation model for MetS on child personal, social, and emotional outcomes (PSE) at 5 years old with cord blood mediators of low-density lipoprotein (LDL), adiponectin (ADI), leptin (LEP), triglycerides (TRI), and high-density lipoprotein levels (HDL) levels. Adjusted for potential covariates of maternal education, maternal age, maternal alcohol intake, maternal smoking, deprivation indices, child sex, ethnicity, gestational age, and birth weight. TE, total effect (direct effect + indirect effect); T-IE, total indirect effect. *p < .05, **p < .01, ***p < .001

**Fig. 5:**
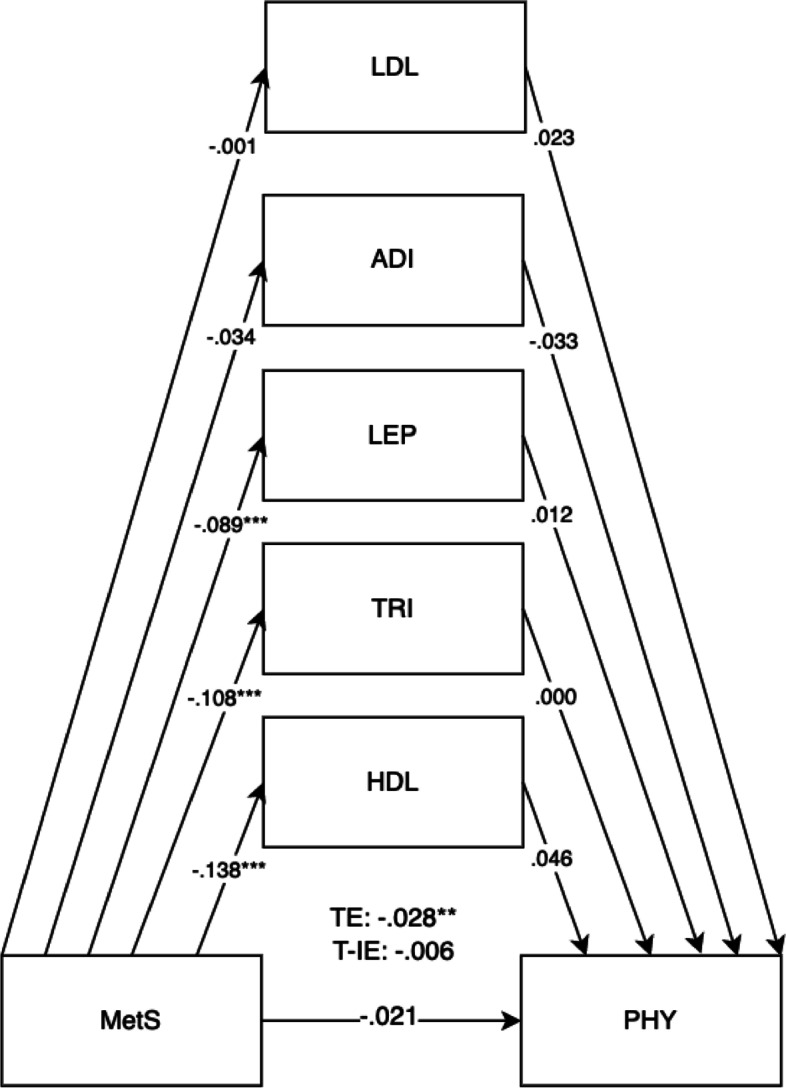
Mediation model for MetS on child physical development outcomes (PHY) at 5 years old with cord blood mediators of low-density lipoprotein (LDL), adiponectin (ADI), leptin (LEP), triglycerides (TRI), and high-density lipoprotein levels (HDL). Adjusted for potential covariates of maternal education, maternal age, maternal alcohol intake, maternal smoking, deprivation indices, child sex, ethnicity, gestational age, and birth weight. TE, total effect (direct effect + indirect effect); T-IE, total indirect effect. *p < .05, **p < .01, ***p < .001

**Fig. 6:**
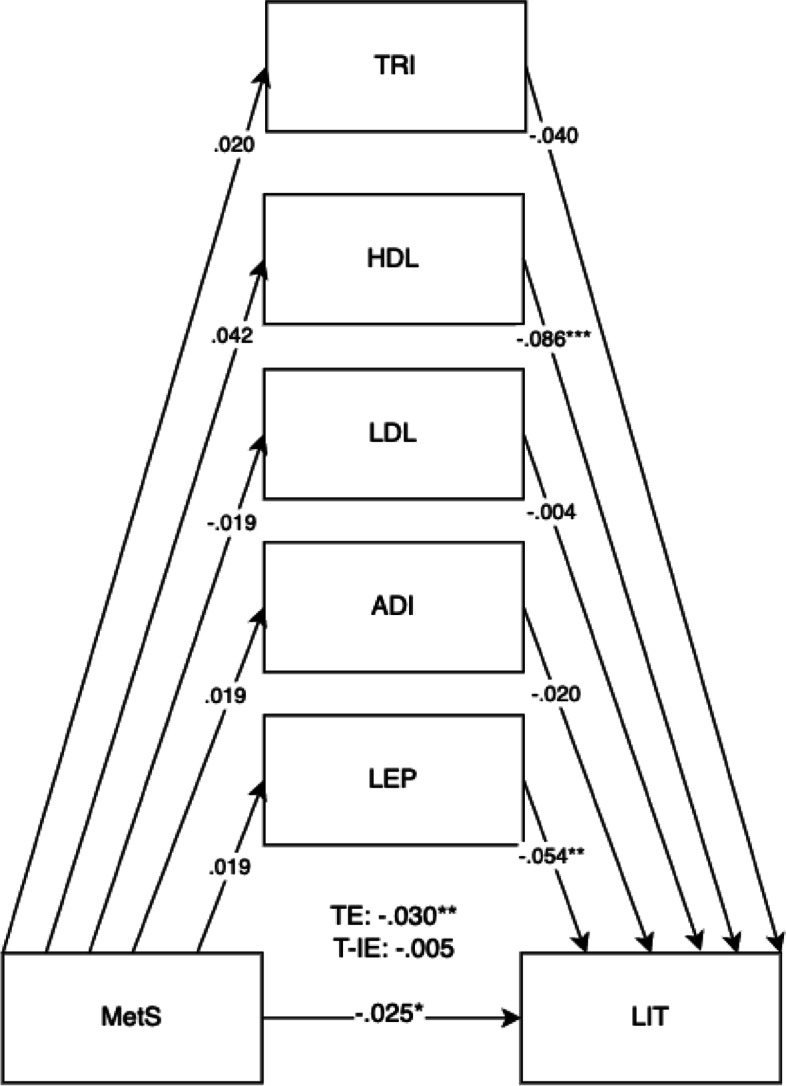
Mediation model for MetS on child literacy (LIT) outcomes at 5 years old with cord blood mediators of low-density lipoprotein (LDL), adiponectin (ADI), leptin (LEP), triglycerides (TRI), and high-density lipoprotein levels (HDL) levels. Adjusted for covariates of maternal education, maternal age, maternal alcohol intake, maternal smoking, deprivation indices, child sex, ethnicity, gestational age, and birth weight. TE, total effect (direct effect + indirect effect); T-IE, total indirect effect. *p < .05, **p < .01, ***p < .001

**Fig. 7:**
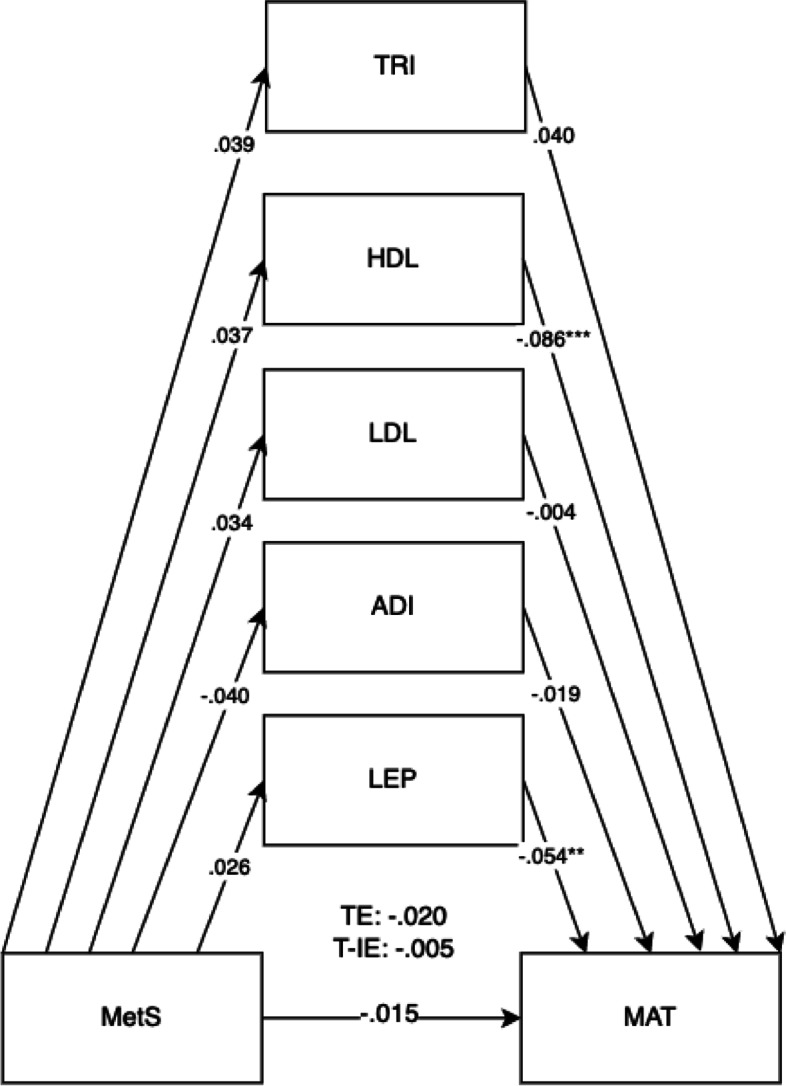
Mediation model for MetS on child mathematics outcomes at 5 years old with cord blood mediators of low-density lipoprotein (LDL), adiponectin (ADI), leptin (LEP), triglycerides (TRI), and high-density lipoprotein levels (HDL) levels. Adjusted for covariates of maternal education, maternal age, maternal alcohol intake, maternal smoking, deprivation indices, child sex, ethnicity, gestational age, and birth weight. TE, total effect (direct effect + indirect effect); T-IE, total indirect effect. *p < .05, **p < .01, ***p < .001

Model fits were evaluated according to fit indices of root mean square error of approximation (RMSEA), standardised root mean square residual (SRMR), comparative fit index (CFI), and Tucker–Lewis index (TLI). Cut-offs were set at < 0.06 for RMSEA and SRMR and ≥ 0.95 for CFI and TLI for judging good fit [32]. Structural equation models were selected due to their advantages for path mediation analysis. This method of analysis was chosen for its flexibility to understand the mediating mechanisms, unlike multiple regressions which requires the running of multiple separate regression models to test for mediation and lower statistical power [33] (Figs. 1, 2, 3, 4, 5, 6 and 7).

## Results

The results in Table 1 showed that 21.1% of mothers had high BMI (≥ 30), 57.9% had raised triglycerides levels (> 1.7 mmol/L), 15.9% with lowered HDL (< 1.6 mmol/L), 1.5% with raised systolic BP (≥ 140), 0.5% with raised diastolic BP (≥ 90), and 3.0% with raised fasting glucose (> 5.6 mmol/L). 0.9% of mothers were previously diagnosed with hypertension, and 3.0% were previously diagnosed with diabetes prior to pregnancy. 4.9% of mothers in this cohort met the IDF classification for maternal metabolic syndrome (Table 1). 45.6% of mothers were Pakistani, 39.6% were White British, and 15.2% were classified as other races (Table 3). Descriptive statistics for predictors, outcomes, and confounders and covariates can be found in Tables 1, 2, and 3. For unadjusted models, sample sizes ranged from *n* = 12,644 to *n* = 13,364 participants. For adjusted models, total sample sizes ranged from *n* = 13,812 to *n* = 13,832 participants, depending on the availability of mother–child pairs for specific models (Table 4).

**Table 4:** Direct, indirect, total indirect, and total effects of child mediators on maternal metabolic syndrome (MetS) on child outcomes (adjusted model)

(a) MetS on	DE	DE 95% CI	IE	IE 95% CI	T-IE	T-IE 95% CI	TE	TE 95% CI
**BPVS (*n* = 13,812)**	.000	− .033 to .036			− .004	− .012 to .004	− .004	− .036 to .031
(a) Triglycerides	− .039	− .093 to .000	.000	− .006 to .002				
(b) Triglycerides	.010	− .057 to .083						
(a) HDL	− .088***	− .124 to − .048	− .005	− .014 to .002				
(b) HDL	.056	− .028 to .139						
(a) LDL	− .002	− .049 to .040	0.00	− .002 to .003				
(b) LDL	− 0.30	− .105 to .042						
(a) Adiponectin	− .019	− .067 to .033	0.00	− .001 to .005				
(b) Adiponectin	− .021	− .083 to .040						
(a) Leptin	− .054**	− .098 to − .026	.001	− .036 to .031				
(b) Leptin	− .014	− .055 to .051						
**LID (*n* = 13,812)**	.010	− .031 to .046			.003	− .003 to .012	.014	− .026 to .047
(a) Triglycerides	− .039	− .092 to − .001	.000	− .004 to .003				
(b) Triglycerides	.000	− .074 to .065						
(a) HDL	− .086***	− .121 to − .046	.003	− .003 to .011				
(b) HDL	− .036	− .109 to .036						
(a) LDL	− .004	− .051 to .039	.000	− .003 to .005				
(b) LDL	− .064	− .138 to − .009						
(a) Adiponectin	− .020	− .066 to 0.33	.000	− .004 to .001				
(b) Adiponectin	.008	− .060 to .068						
(a) Leptin	− .054**	− .098 to − .026	.000	− .002 to .002				
(b) Leptin	− .005	− .042 to .060						
**COM (*n* = 13,832)**	− .011	− .032 to .012			− .007*	− .014 to − .001	− .018	− .038 to .004
(a) Triglycerides	− .040	− .092 to .004	− .001	− .006 to .001				
(b) Triglycerides	.025	− .025 to .076						
(a) HDL	− .086***	− .121 to − .046	− .005	− .012 to .000				
(b) HDL	.059	.002 to .118						
(a) LDL	− .004	− .051 to .040	.000	− .003 to .002				
(b) LDL	.035	− .025 to .076						
(a) Adiponectin	− .020	− .072 to .028	.001	− .001 to .004				
(b) Adiponectin	− .032	− .081 to .018						
(a) Leptin	− .054**	− .097 to − .027	− .001	− .003 to .000				
(b) Leptin	.019	− .010 to .055						
**PSE (*n* = 13,832)**	− .007	− .027 to .016			− .006*	− .013 to − .001	− .013	− .032 to .007
(a) Triglycerides	− .040	− .092 to .004	− .001	− .005 to .001				
(b) Triglycerides	.018	− .030 to .063						
(a) HDL	− .087***	− .121 to − .047	− .005	− .013 to − .001				
(b) HDL	.062	.003 to .120						
(a) LDL	− .004	− .051 to .040	.000	− .003 to .001				
(b) LDL	.032	− .023 to .085						
(a) Adiponectin	− .019	− .072 to .026	.000	− .001 to .004				
(b) Adiponectin	− .023	− .069 to .029						
(a) Leptin	− .054**	− .097 to − .027	.000	− .032 to .002				
(b) Leptin	.003	− .025 to .039						
**PHY (*n* = 13,832)**	− .011	− .032 to .011			− .005	− .012 to .000	− .015	− .036 to − .005
(a) Triglycerides	− .039	− .091 to .004	− .000	− .003 to .002				
(b) Triglycerides	.002	− .045 to .050						
(a) HDL	− .087***	− .121 to − .046	− .005	− .011 to − .000				
(b) HDL	.054	− .001 to .111						
(a) LDL	− .004	− .050 to .041	.000	− .003 to .001				
(b) LDL	.027	− .026 to .079						
(a) Adiponectin	− .019	− .071 to .029	.001	− .001 to .004				
(b) Adiponectin	− .036	− .085 to .011						
(a) Leptin	− .054**	− .097 to − .027	− .001	− .003 to .001				
(b) Leptin	.010	− .019 to .043						
**LIT (*n* = 13,832)**	− .025*	− .045 to − .005			− .005	− .011 to .000	− .030**	− .051 to − .010
(a) Triglycerides	− .040	− .092 to .004	− .001	− .005 to .001				
(b) Triglycerides	.020	− .029 to .067						
(a) HDL	− .086***	− .121 to − .046	− .004	− .010 to .001				
(b) HDL	.042	− .010 to .099						
(a) LDL	− .004	− .052 to .040	.000	− .003 to .001				
(b) LDL	.033	− .019 to .084						
(a) Adiponectin	− .020	− .070 to .027	.000	− .001 to .003				
(b) Adiponectin	− .019	− .067 to .031						
(a) Leptin	− .054**	− .097 to − .026	− .001	− .003 to .000				
(b) Leptin	.019	− .009 to .055						
**MAT (*n* = 13,832)**	− .015	− .035 to .008			− .005	− .012 to .000	− .020	− .040 to .001
(a) Triglycerides	− .040	− .092 to .004	− .002	− .006 to .000				
(b) Triglycerides	.039	− .007 to .084						
(a) HDL	− .086***	− .121 to − .047	− .003	− .009 to .001				
(b) HDL	.037	− .015 to .092						
(a) LDL	− .004	− .052 to .040	.000	− .003 to .001				
(b) LDL	.034	− .023 to .083						
(a) Adiponectin	− .019	− .071 to .028	.001	− .001 to .004				
(b) Adiponectin	− .040	− .089 to .010						
(a) Leptin	− .054**	− .097 to − .027	− .001	− .004 to .000				
(b) Leptin	.026	− .004 to .062						

Participant sample sizes ranged from 12,652 (found in unadjusted models) to 13,832 mother–child pairs depending on the availability of data

*DE* direct effect, *IE* indirect effect, *T-IE* total indirect effect, *TE* total effects (direct effect + indirect effect)

(a) Child mediators on METSYN; (b) child outcomes on child mediators

^*^p < .05, ^**^p < .01, ^***^p < .001

The mediation models showed adequate fit according to the fit indices (CFI > 0.90, TLI > 0.80, RMSEA = 0.21, SRMR = 0.13). All model fits can be found in Additional file 1: Table S1. Statistical significance was set at *p* < 0.05.

The overall results for the adjusted model found the total effects of maternal metabolic syndrome through child mediators on the domains of LIT (*B* =  − 0.030, 95% CI =  − 0.046, − 0.014).

Direct effects were found for maternal metabolic syndrome on the LIT domain (*B* =  − 0.025, 95% CI =  − 0.041, − 0.008). Direct effects for maternal metabolic syndrome were also shown for child mediators of HDL (*B* =  − 0.086, 95% CI =  − 0.117, − 0.052) and leptin (*B* =  − 0.054, 95% CI =  − 0.089, − 0.032) in all child development domains. The pathway of effect shows that the effects of all combined child cord blood markers acted as mediators for maternal metabolic syndrome and child development outcomes, showing total indirect effects. Total indirect effects were found for the COM (*B* =  − 0.007, 95% CI =  − 0.012, − 0.002) and PSE domains (*B* =  − 0.006, 95% CI =  − 0.012, − 0.002). More specific results can be found in Table 4.

The mediation proportion has also been calculated as follows: for the BPVS domain = 100%, for the LID domain = 21.4%, for the COM domain = 38.8%, for the PSE domain = 46.2%, for the PHY domain = 33.3%, for the LIT domain = 16.7%, and for the MAT domain = 25%.

A sensitivity analysis was run with added covariates of gestational diabetes, gestational hypertension, and preeclampsia. When including these additional covariates, results stayed essentially the same as the main analysis. Consistent associations were found for the total effects of maternal metabolic syndrome through child mediators on the domains of LIT (*B* =  − 0.033, 95% CI =  − 0.051, − 0.011).

More information on both the unadjusted and adjusted models can be found in Table 4, Additional file 1: S1-S5, or on OSF [25].

## Discussion

The purpose of the present study was to examine child cardiometabolic markers as potential mediators of the links between maternal metabolic syndrome in pregnancy and child developmental outcomes at 5 years old. We found that there were significant total effects of maternal metabolic syndrome on child developmental outcomes in the literacy domain (LIT) at age 5 both before and after adjustments for covariates. Maternal metabolic syndrome was found to affect specific child cord blood markers of lowered HDL and increased leptin levels, displaying these two markers as potential at-risk cord blood biomarkers at birth when maternal metabolic syndrome is present during pregnancy when assessing children’s literacy outcomes. Total indirect effects also showed that the combined effects of an at-risk profile at birth (using all child cord blood markers) partially mediated the association between maternal cardiometabolic health and children’s communication and language (COM) and personal, social, and emotional (PSE) domains at 5 years old.

The above results support the hypotheses of maternal cardiometabolic health being associated with developmental outcomes at 5 years old. Additionally, while the effects of individual cord blood markers were too small to be individually significant, the direct and indirect effects of all combined cord blood markers (HDL, LDL, triglycerides, adiponectin, and leptin) showed significant mediating effects for maternal metabolic syndrome and child development domains of COM and PSE. The study results were in line with a study using two pregnancy cohorts, where some cord blood markers were seen to be associated with child neurodevelopment at age 3, 5, and 8 years old, specifically for performance and full-scale IQ, and working memory [26]. While the previous study only looked at hormones of adiponectin and leptin individually, this study adds to the literature by allowing for a closer examination of the combined effects of multiple markers to be studied together.

The study results were also consistent with the literature on maternal cardiometabolic conditions affecting both cord blood profiles [34] and neurodevelopment. Overall findings were in line with research on low-grade, chronic inflammatory processes such as cardiometabolic health risk in the mother during pregnancy being linked with foetal development [35] and subsequent effects on outcomes such as IQ, socialisation, communication, expressive language, physical development, and executive function [8, 9, 36, 37]. Closer examination of the results showed evidence for complex underlying mechanisms of maternal cardiometabolic profiles being linked with broader domains commonly assessed through education at a starting-school age, such as communication, literacy, and physical development, even when accounting for maternal, environmental, and child covariates and confounders.

Using the classification of metabolic syndrome allowed the examination of the combined effects of multiple biomarkers. Diabetes and hypertension seem to share similar processes of oxidative stress-mediated regulation cascades and chronic, low-grade inflammation [38] with the release of free fatty acids from adipose tissue being linked with higher oxidative stress and maternal endothelial dysfunction [39]. With this increased metabolic demand and homeostatic disruption during pregnancy, studies found effects on foetal outcomes, in birth or childhood cardiovascular outcomes over time [40–42]. In addition, the cord blood marker of lowered HDL, also classified as an oxidative stress marker [43], presents an early insight into how it can affect a child’s personal, social, and emotional domain, with studies supporting associations when examining oxidative stress dysregulation being found in patients with depression and even subsequent suicide attempts [44, 45]. The current study results are supported by previous research and add a further dimension, that is, it found that combined effects of lowered child health at birth mediated the negative effects of maternal metabolic syndrome on development. This is consistent with the inflammatory cascade hypothesis that postulates inflammation during pregnancy affecting not only early-life programming, but also subsequently showing negative effects on child development [46].

### Study implications

This study demonstrates how maternal cardiometabolic health may contribute to later child developmental difficulties. Maternal metabolic syndrome classification, a constellation of metabolic risk factors, seems to not only affect the pregnancy process but is also linked with specific developmental delays in communication, literacy, and personal, social, and emotional development at 5 years old. This is a critical period for learning and subsequent educational attainment.

Moreover, this study further adds to the literature on gestational biology mechanisms not only affecting foetal health, but also a newborn’s health, and essential child developmental milestones. It is often difficult to justify early clinical intervention for children with no clear prognostic factor during pregnancy. This research provides a theoretical basis for interventions even before developmental delays are formally identified in the child through considering maternal metabolic syndrome classification during pregnancy to be a risk marker when considering child development outcomes. A clinical implication from our findings showed that while both maternal and child cardiometabolic markers seemed statistically non-significant when examined individually, they were found to be significant when examined together. This displays the importance of not looking for excessive values in any one marker, but to examine both a mother and child’s cardiometabolic health as a combined profile when assessing future risk. Furthermore, the results imply that health and educational organisations should be better informed of these potential long-term effects of maternal cardiometabolic health on child developmental milestones. In particular, the start of formal education can possibly consider having extra screening or monitoring systems for affected children at future risk for having special education needs.

### Strengths and limitations

Study strengths include using a large, high-quality, and ethnically diverse cohort sample. A comprehensive set of cardiometabolic markers was taken from clinical data collected during pregnancy. Furthermore, the classification of metabolic syndrome using a standardised classification according to the International Diabetes Federation definition allows for future replicability. While some studies [47, 48] have described associations between pregnancy cardiometabolic markers and childhood health-related outcomes, it still remains unclear whether the associations are predominantly due to intrauterine mechanisms or other external confounders such as birth outcomes or socioeconomic levels. The mediation models in this study provides a closer examination of maternal-child associations with added mediators before any environmental influence.

This study had several limitations. First, this prospective cohort had a limited sample size that met the requirement for metabolic syndrome classification (*n* = 537), in addition to missing data for the child cord blood markers when comparing mother–child pairs. In addition, the measure of diabetes taken by the study team did not differentiate between type I and type II diabetes in the mother. Maternal gestational diabetes was also not included as part of the analysis, due to possible epigenetic aetiology [49]. Next, maternal physical activity, a possible protective mechanism that might mitigate the effect of diabetes and hypertension, was not included as a possible study confounder as there was no standardised measure available from the dataset. Data on specific medication use for clinical management of maternal metabolic syndrome was not collected by the research team and was therefore not included as a potential study factor. Obesity-susceptible single nucleotide polymorphisms (SNP), a component of persistent central obesity and metabolic syndrome, also not included as a study factor that could possibly lead to adverse childhood outcomes [50]. While data was taken from a more diverse area, results may not be reflective of pregnancy cohorts from lower-income countries. Generally, the effect sizes were small for this study. However, indirect effects in the context of mediation analysis are likely to be small and may still be important to consider in the context of population health [51].

## Conclusions

Maternal cardiometabolic risk during pregnancy is associated with child developmental outcomes and newborn health examined through cord blood markers partially mediates this. These findings held after accounting for external variables to distinguish between the direct impact of gestational biology and indirect environmental influence by controlling for maternal, child, and environmental factors. Study outcomes were specifically selected as they are part of a nationwide framework that assesses developmental milestones. These findings highlight a public health need to further understand the mechanistic pathways in which maternal health during pregnancy affects children’s health and subsequent development, which further support preventative or interventions as early as possible. As shown in this study, child cord blood markers are associated with children’s development. Healthcare services can therefore consider adding them as prognostic risk biomarkers as part of continuing clinical care. Study findings also indicate a need for early monitoring in children born to mothers with metabolic syndrome classification during pregnancy due to a higher at-risk profile both at birth (seen in cord blood) and age 5 development. More research needs to be done to further understand if these associations are affected by the presence of other pregnancy risk mechanisms and if developmental outcomes persist beyond 5 years old.

Future research could include a closer examination of ethnic differences, epigenetic effects, or additional childhood factors such as feeding or sleeping and environmental factors contributing to maternal cardiometabolic health and child developmental outcomes. Future research should also look at trimester-based risk for further understanding of inflammation processes when intrauterine stress is present during early, mid, or late pregnancy [52]. 

## Supplementary Information

**Additional file 1: Table S1.** Model fits for models fully adjusted for confounders and covariates. **Table S2.** Direct, indirect, and total effects of child mediators on MetS on child outcomes for unadjusted models. **Table S3.** Correlation table for individual child cardiometabolic markers. **Table S4.** Correlation table for study covariates. **Table S5.** Data collection details by the Born in Bradford study team.

## Data Availability

The datasets generated and analysed during the current study are available in the Born in Bradford repository upon approval from the Born in Bradford team, https://borninbradford.nhs.uk/research/how-to-access-data/.

## Abbreviations

ADI: Adiponectin
BMI: Body mass index
BPVS: British Picture Vocabulary Scale
COM: Communication and language
EGIR: European Group for the Study of Insulin Resistance
EYFS: Early Years Foundation Stage
HDL: High-density lipoprotein
IDF: International Diabetes Federation
LDL: Low-density lipoprotein
LEP: Leptin
LID: Letter Identification Assessment
LIT: Literacy
MAT: Mathematics
PHY: Physical development
PSE: Personal, social, and emotional
SNP: Single nucleotide polymorphisms
TRI: Triglycerides
WHO: World Health Organization
