# Supplementary material for: Maternal metabolic syndrome in pregnancy and child development at age 5: exploring mediating mechanisms using cord blood markers
Source: BMC Med. 2023 Apr 3;21:124. doi: 10.1186/s12916-023-02835-5 (PMC10071709; doi:10.1186/s12916-023-02835-5)
Supplement: Supplementary file 1 — Additional file 1: Table S1. Model fits for models fully adjusted for confounders and covariates. Table S2. Direct, indirect, and total effects of child mediators on MetS on child outcomes for unadjusted models. Table S3. Correlation table for individual child cardiometabolic markers. Table S4. Correlation table for study covariates. Table S5. Data collection details by the Born in Bradford study team. [file 12916_2023_2835_MOESM1_ESM.docx]

Additional file 1

Table S1. Model Fits (adjusted models)

|  | **n** | **CFI** | **TLI** | **RMSEA** | **SRMR** |
| --- | --- | --- | --- | --- | --- |
| **BVPS** | 13812 | 0.985 | 0.826 | 0.021 | 0.013 |
| **LID** | 13812 | 0.984 | 0.812 | 0.021 | 0.013 |
| **COM** | 13832 | 0.987 | 0.851 | 0.021 | 0.013 |
| **PSE** | 13832 | 0.987 | 0.847 | 0.021 | 0.013 |
| **PHY** | 13832 | 0.987 | 0.847 | 0.021 | 0.013 |
| **LIT** | 13832 | 0.987 | 0.854 | 0.021 | 0.013 |
| **MAT** | 13832 | 0.987 | 0.845 | 0.021 | 0.013 |

Table S2. Direct, indirect, and total effects of child mediators on MetS on child outcomes (unadjusted model)

| 1. MetS on | **DE** | **DE *95% CI*** | **IE** | **IE *95% CI*** | **T-IE** | **T-IE 95% CI** | **TE** | **TE *95% CI*** |
| --- | --- | --- | --- | --- | --- | --- | --- | --- |
| **BPVS (n=12646)** | .005 | -.029 to .035 |  |  | -.005 | -.014 to .003 | .000 | -.031 to .029 |
| (a) Triglycerides | -.006 | -.056 to .038 | .000 | -.001 to .004 |  |  |  |  |
| (b) Triglycerides | -.027 | -.093 to .049 |  |  |  |  |  |  |
| (a) HDL | -.075*** | -.111 to -.034 | -.006 | -.015 to .000 |  |  |  |  |
| (b) HDL | .073 | -.008 to .157 |  |  |  |  |  |  |
| (a) LDL | -.015 | -.061 to .024 | .000 | -.001 to .005 |  |  |  |  |
| (b) LDL | -.023 | -.092 to .040 |  |  |  |  |  |  |
| (a) Adiponectin | -.012 | -.054 to .038 | .000 | -.001 to .003 |  |  |  |  |
| (b) Adiponectin | -.008 | -.070 to .057 |  |  |  |  |  |  |
| (a) Leptin | -.089*** | -.139 to -.053 | .000 | -.004 to .003 |  |  |  |  |
| (b) Leptin | .003 | -.037 to .047 |  |  |  |  |  |  |
| **LID (n= 12644)** | .008 | -.029 to .047 |  |  | .004 | -.004 to .011 | .012 | -.025 to .049 |
| (a) Triglycerides | -.006 | -.061 to .039 | .000 | -.001 to .003 |  |  |  |  |
| (b) Triglycerides | -.005 | -.073 to .057 |  |  |  |  |  |  |
| (a) HDL | -.074*** | -.113 to -.028 | .000 | -.006 to .006 |  |  |  |  |
| (b) HDL | .006 | -.078 to .075 |  |  |  |  |  |  |
| (a) LDL | -.016 | -.060 to .023 | .001 | -.001 to .006 |  |  |  |  |
| (b) LDL | -.058 | -.130 to .012 |  |  |  |  |  |  |
| (a) Adiponectin | -.012 | -.060 to .023 | .000 | -.004 to .001 |  |  |  |  |
| (b) Adiponectin | .014 | -.050 to .081 |  |  |  |  |  |  |
| (a) Leptin | -.089*** | -.142 to -.054 | .004* | .001 to .008 |  |  |  |  |
| (b) Leptin | -.043* | -.094 to -.016 |  |  |  |  |  |  |
| **COM (n= 13364)** | -.017 | -.038 to .005 |  |  | -.006 | -.013 to .001 | -.022* | -.042 to -.002 |
| (a) Triglycerides | -.007 | -.061 to .037 | .000 | -.003 to .001 |  |  |  |  |
| (b) Triglycerides | .014 | -.045 to .059 |  |  |  |  |  |  |
| (a) HDL | -.075*** | -.114 to .034 | -.008* | -.015 to -.003 |  |  |  |  |
| (b) HDL | .101*** | .046 to .158 |  |  |  |  |  |  |
| (a) LDL | -.016 | -.065 to .025 | .000 | -.004 to .001 |  |  |  |  |
| (b) LDL | .028 | -.027 to .083 |  |  |  |  |  |  |
| (a) Adiponectin | -.012 | -.060 to .039 | .000 | -.001 to .003 |  |  |  |  |
| (b) Adiponectin | -.009 | -.061 to .040 |  |  |  |  |  |  |
| (a) Leptin | -.089*** | -.147 to -.056 | .002* | .000 to .005 |  |  |  |  |
| (b) Leptin | -.028* | -.055 to -.004 |  |  |  |  |  |  |
| **PSE (n= 13364)** | -.014 | -.035 to .007 |  |  | -.005 | -.012 to .002 | -.018 | -.039 to -.001 |
| (a) Triglycerides | -.007 | -.060 to .037 | .000 | -.002 to .001 |  |  |  |  |
| (b) Triglycerides | .006 | -.038 to .052 |  |  |  |  |  |  |
| (a) HDL | -.075*** | -.114 to .033 | -.008* | -.015 to -.003 |  |  |  |  |
| (b) HDL | .102*** | .046 to .156 |  |  |  |  |  |  |
| (a) LDL | -.016 | -.064 to .025 | .000 | -.004 to .001 |  |  |  |  |
| (b) LDL | .025 | -.033 to .079 |  |  |  |  |  |  |
| (a) Adiponectin | -.012 | -.062 to .039 | .000 | -.001 to .002 |  |  |  |  |
| (b) Adiponectin | -.004 | -.051 to .045 |  |  |  |  |  |  |
| (a) Leptin | -.089*** | -.147 to -.057 | .003* | .001 to .006 |  |  |  |  |
| (b) Leptin | -.037** | -.068 to -.014 |  |  |  |  |  |  |
| **PHY (n= 13364)** | -.018 | -.039 to .004 |  |  | -.003 | -.010 to .003 | -.021 | -.041 to .000 |
| (a) Triglycerides | -.007 | -.060 to .037 | .000 | -.001 to .002 |  |  |  |  |
| (b) Triglycerides | -.008 | -.056 to .041 |  |  |  |  |  |  |
| (a) HDL | -.075*** | -.114 to -.033 | -.007* | -.014 to -.003 |  |  |  |  |
| (b) HDL | .095*** | .041 to .146 |  |  |  |  |  |  |
| (a) LDL | -.016 | -.064 to .025 | .000 | -.003 to .001 |  |  |  |  |
| (b) LDL | .019 | -.040 to .074 |  |  |  |  |  |  |
| (a) Adiponectin | -.012 | -.060 to .039 | .000 | -.001 to .003 |  |  |  |  |
| (b) Adiponectin | -.018 | -.064 to .029 |  |  |  |  |  |  |
| (a) Leptin | -.089*** | -.147 to -.056 | .004** | .001 to .007 |  |  |  |  |
| (b) Leptin | -.043** | -.074 to -.018 |  |  |  |  |  |  |

| **LIT (n= 13364)** | -.033** | -.053 to -.013 |  |  | -.005 | -.012 to .001 | **-**.037*** | -.057 to -.018 |
| --- | --- | --- | --- | --- | --- | --- | --- | --- |
| (a) Triglycerides | -.007 | -.060 to .037 | .000 | -.003 to .001 |  |  |  |  |
| (b) Triglycerides | .018 | -.040 to .060 |  |  |  |  |  |  |
| (a) HDL | -.075*** | -.114 to -.032 | -.007* | -.015 to -.002 |  |  |  |  |
| (b) HDL | .089** | .033 to .145 |  |  |  |  |  |  |
| (a) LDL | -.016 | -.064 to .026 | .000 | -.004 to .001 |  |  |  |  |
| (b) LDL | .027 | -.031 to .082 |  |  |  |  |  |  |
| (a) Adiponectin | -.013 | -.062 to .038 | .000 | -.001 to .002 |  |  |  |  |
| (b) Adiponectin | -.005 | -.056 to .041 |  |  |  |  |  |  |
| (a) Leptin | -.089*** | -.147 to -.056 | .003* | .001 to .006 |  |  |  |  |
| (b) Leptin | -.030* | -.057 to -.008 |  |  |  |  |  |  |
| **MAT (n= 13364)** | -.021 | -.042 to -.001 |  |  | -.005 | -.012 to .001 | -.025* | -.047 to -.006 |
| (a) Triglycerides | -.007 | -.059 to .037 | .000 | -.003 to .001 |  |  |  |  |
| (b) Triglycerides | .031 | -.019 to .075 |  |  |  |  |  |  |
| (a) HDL | -.075*** | -.114 to -.033 | -.005* | -.013 to -.001 |  |  |  |  |
| (b) HDL | .071* | .017 to .127 |  |  |  |  |  |  |
| (a) LDL | -.017 | -.065 to .025 | -.001 | -.005 to .001 |  |  |  |  |
| (b) LDL | .036 | -.017 to .091 |  |  |  |  |  |  |
| (a) Adiponectin | -.012 | -.061 to .038 | .000 | -.001 to .003 |  |  |  |  |
| (b) Adiponectin | -.025 | -.073 to .022 |  |  |  |  |  |  |
| (a) Leptin | -.089*** | -.147 to -.056 | .001 | .001 to .004 |  |  |  |  |
| (b) Leptin | -.014 | -.039 to .010 |  |  |  |  |  |  |

*p<.05*, p<.01*, p<.001**

***DE****: Direct Effect*

***IE:*** *Indirect Effect*

***T-IE:*** *Total Indirect Effect*

***TE:*** *Total Effects (Direct Effect + Indirect Effect)*

*(a)Child mediators on MetS; (b) Child outcomes on child mediators*

*Note: participant sample sizes ranged from 12,652 to 13,832 (found in adjusted models) mother-child pairs depending on availability of data*

Correlations

Table S3. Correlation Table for Child Cardiometabolic Markers

|  |  | **1** | **2** | **3** | **4** | **5** |
| --- | --- | --- | --- | --- | --- | --- |
| **1** | **Triglycerides** | 1 |  |  |  |  |
| **2** | **HDL** | .234*** | 1 |  |  |  |
| **3** | **LDL** | .012 | -.452*** | 1 |  |  |
| **4** | **Adiponectin** | .036 | -.037 | .051* | 1 |  |
| **5** | **Leptin** | .022 | -.078 | -.081* | .117*** | 1 |

*p<.05*, p<.01**, p<.001****

Table S4. Correlation Table for Covariates

|  |  | **1** | **2** | **3** | **4** | **5** | **6** | **7** | **8** |
| --- | --- | --- | --- | --- | --- | --- | --- | --- | --- |
| **1** | **Maternal Education** | 1 |  |  |  |  |  |  |  |
| **2** | **Maternal Age** | .193*** | 1 |  |  |  |  |  |  |
| **3** | **Maternal Alcohol** | .028** | -.040*** | 1 |  |  |  |  |  |
| **4** | **Maternal Smoking** | -.193*** | -.221*** | .286*** | 1 |  |  |  |  |
| **5** | **Deprivation Indices** | .193*** | .165*** | .274*** | -.036 | 1 |  |  |  |
| **6** | **Child Sex** | .017 | .008 | .016 | .009*** | .005 | 1 |  |  |
| **7** | **Gestation** | .022* | -.068*** | .051*** | -.013*** | .022 | .022 | 1 |  |
| **8** | **Birthweight** | .053*** | .044*** | .138*** | -.045*** | .096*** | .096 | .618*** | 1 |

*p<.05*, p<.01**, p<.001****


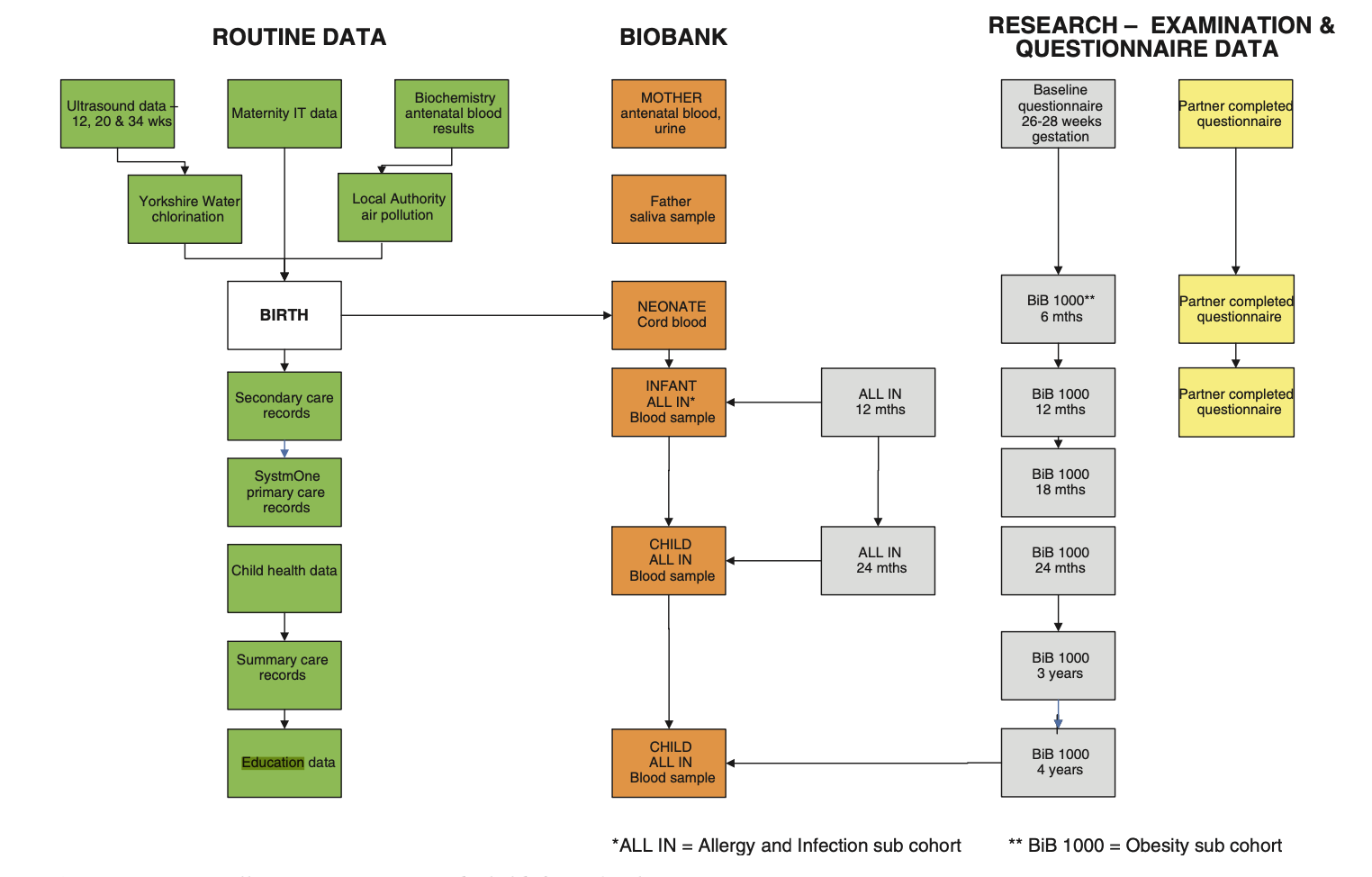


Participant flowchart from BIB research team (55).

**Note:** Reliability testing of the growth data measurements by BiB health workers indicated good quality control for inter- and intra-observer technical error of measurements (r=0.96-1.00) (56).

Table S5. Data Collection Details.

| **Variables** | **Collection Details** |
| --- | --- |
| **Demographic data** |  |
| Singleton births (n=13455) | Medical record |
| Twins (n=354; 177 sets) | Medical record |
| Triplets (n=9; 3 sets) | Medical record |
| **Child Biological Samples** |  |
| Child cord blood markers (Assay) | 13ml; from medical data as extracted from the eCLipse maternity IT system, taken at birth. More details can be found here: https://academic.oup.com/ije/article/42/4/978/655870#supplementary-data |
| **Mother Biological Samples (taken at 28 weeks)** |  |
| Body Mass Index (BMI) | Weighed and measured at time of recruitment (28 weeks) |
| Systolic blood pressure | From baseline measure at 28 weeks, taken by clinical staff |
| Diastolic blood pressure | From baseline measure at 28 weeks, taken by clinical staff |
| Fasting Glucose | From baseline measure at 28 weeks; 13 mls blood sample, prepared for storage at -80°C: serum, whole blood, plasma, buffy coat and red blood cells |
| HDL Cholesterol | From baseline measure at 28 weeks; 13 mls blood sample, prepared for storage at -80°C: serum, whole blood, plasma, buffy coat and red blood cells |
| Triglycerides | From baseline measure at 28 weeks; 13 mls blood sample, prepared for storage at -80°C: serum, whole blood, plasma, buffy coat and red blood cells |
| Previously diagnosed diabetes | From health services data collected as part of clinical care |
| Gestational diabetes | Baseline measure, maternity data set; diagnosed by glucose tolerance test based on WHO (WHO/NCD/NCS/99.2) thresholds for impaired glucose tolerance or impaired fasting glucose, (i.e. fasting plasma glucose ≥6.0mmol/l and/or 2-hr post-challenge glucose ≥ 7.8mmol/l) at 26 weeks. |
| **Sociodemographic characteristics** |  |
| Deprivation | Taken by research team at baseline questionnaire (28 weeks) |
| Education - Mother | Taken by research team at baseline questionnaire (28 weeks) |
| **Self-reported health behaviour** |  |
| Smoking Status | Self-reported by mothers throughout pregnancy |
| Mental Health | Self-reported by mothers throughout pregnancy |
